# Dyspnea After an Acute Intermediate-Risk Pulmonary Embolism: A Case-Based Approach to Evaluation and Treatment

**DOI:** 10.1016/j.jaccas.2024.102540

**Published:** 2024-09-18

**Authors:** Brooke Zlotshewer, Estefania Oliveros, Zachary Meilli, Amine Nasri, Anjali Vaidya, Vladimir Lakhter, Ahmed S. Sadek, Paul Forfia, Riyaz Bashir

**Affiliations:** a Department of Medicine, Lewis Katz School of Medicine at Temple University, Philadelphia, Pennsylvania, USA; b Division of Cardiovascular Disease, Lewis Katz School of Medicine at Temple University, Philadelphia, Pennsylvania, USA

**Keywords:** chronic thromboembolic pulmonary disease, chronic thromboembolic pulmonary hypertension, pulmonary embolism, residual pulmonary vascular obstruction

## Abstract

Exercise intolerance after acute pulmonary embolism may be caused by residual pulmonary vascular obstruction, which presents as chronic thromboembolic pulmonary disease with or without pulmonary hypertension. We present a case highlighting a systematic approach to evaluating functional limitations due to residual pulmonary vascular obstruction, emphasizing the utility of cardiopulmonary exercise testing.

## Graphical Abstract

**Figure:**
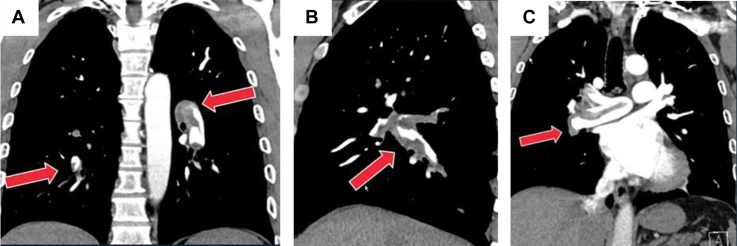

## Case Presentation

**Figure 1:**
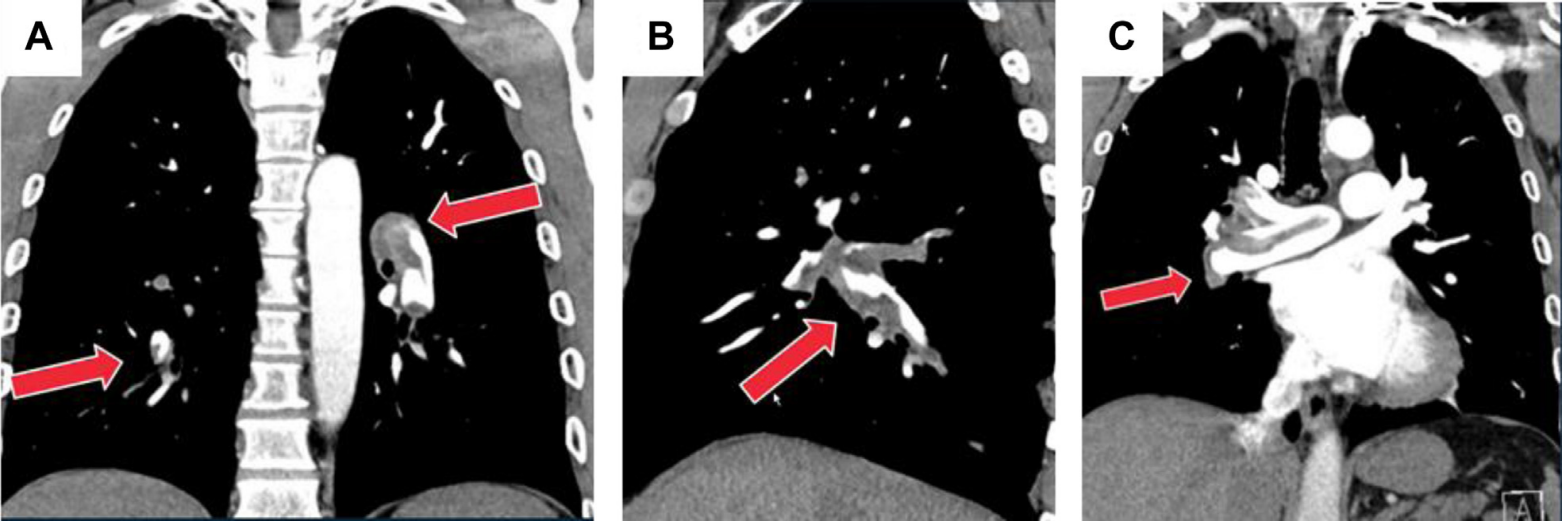
Acute Intermediate-Risk Pulmonary Embolism Saddle embolism involving the right and left pulmonary arteries.

A 56-year-old physically active man presented to Temple University Hospital with exercise intolerance during high-intensity exercise (ie, midfielder soccer player, hiking). Past medical history included travel-related acute deep vein thrombosis and acute intermediate-risk pulmonary embolism (PE) 1 year before the current presentation (Figure 1). He was treated with anticoagulation alone, followed by 3 months of dabigatran; his hypercoagulable work-up was negative. He had no further limitations with routine activities; however, he noted residual dyspnea when playing soccer.

**Learning Objectives**

• To identify residual pulmonary vascular obstruction in a patient with a history of PE with a normal echocardiogram.
• To understand the role of CPET in patients with post-PE impairment.
• To highlight the utility of BPA in patients with CTEPD and post-PE impairment.

**Figure 2:**
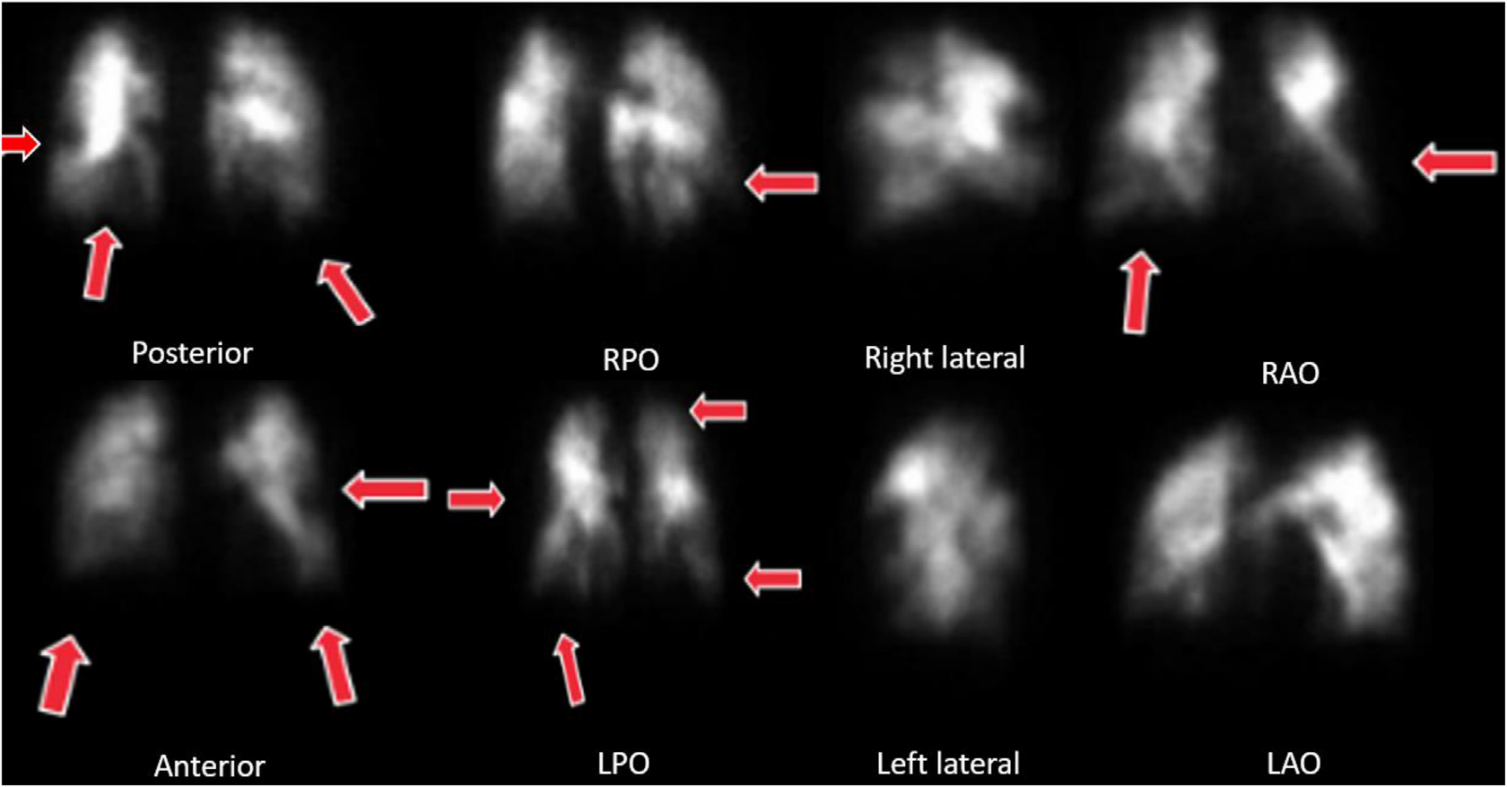
Lung Perfusion Scan With Bilateral Multiple Moderate to Large Perfusion Defect All pressures are reported in mm Hg. CI reported in L/min/m^2^. CO reported in L/min. BPA = balloon pulmonary angioplasty; CI = cardiac index; CO = cardiac output; PAP = pulmonary arterial pressure; PVR = pulmonary vascular resistant reported in Wood Units; SVO_2_ = venous oxygen saturation.

An exercise stress test was negative for myocardial ischemia but revealed hypoxemia (85%) at peak exercise. An echocardiogram demonstrated normal biventricular size and function without features of pulmonary hypertension (PH). A lung perfusion scan showed multiple bilateral wedge-shaped perfusion defects (Figure 2). His 6-minute walking distance test (6MWD) was 531 m. Computed tomography angiography scan of the chest demonstrated features of chronic thromboembolic pulmonary disease (CTEPD) within the segmental and subsegmental pulmonary arteries.

**Figure 3:**
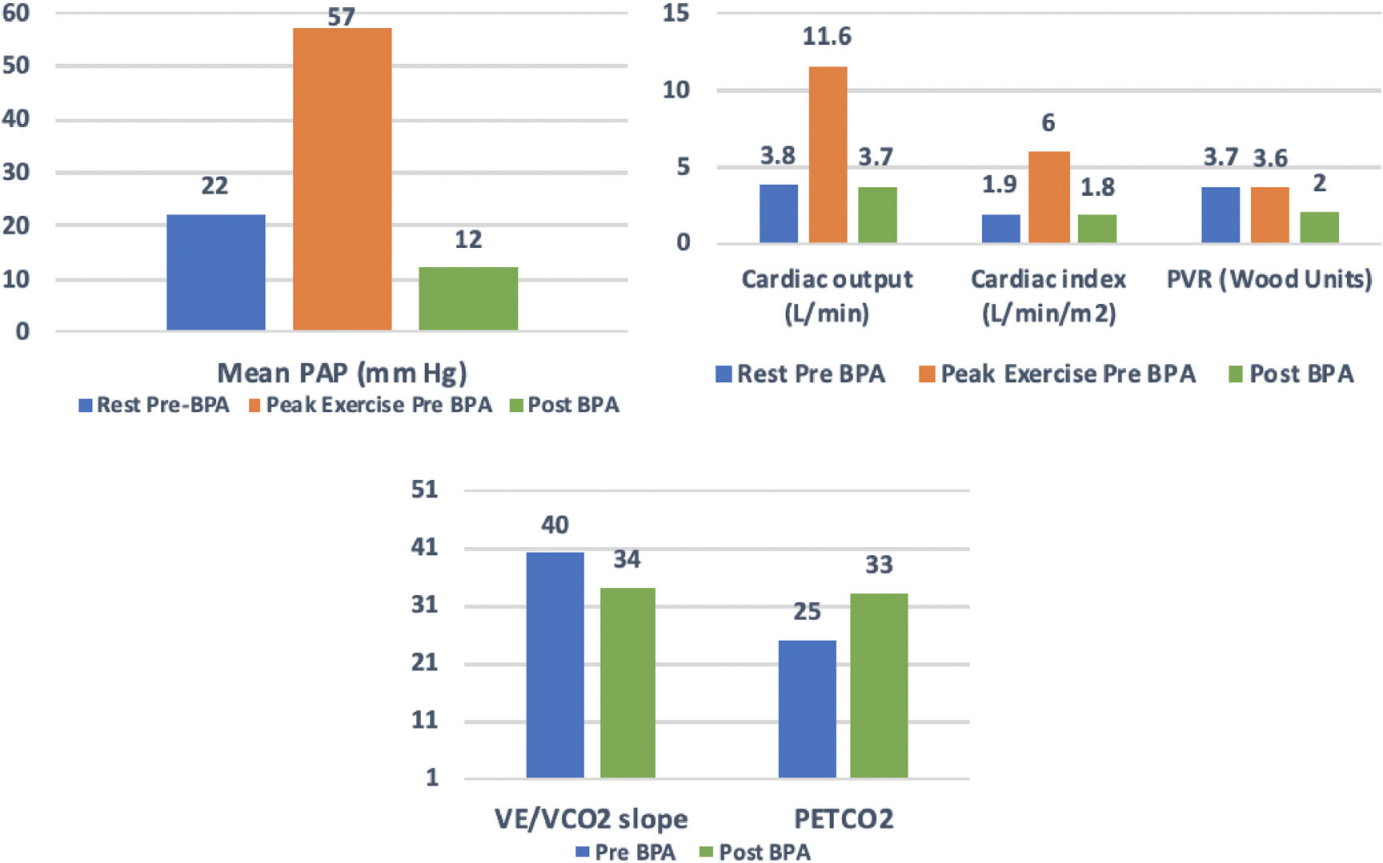
Hemodynamic Changes During Invasive Cardiopulmonary Exercise Testing

Resting hemodynamics revealed a right atrial (RA) pressure of 1 mm Hg, pulmonary artery pressure (PAP) of 35/15 (22) mm Hg, pulmonary capillary wedge pressure of 8 mm Hg, cardiac output (CO) of 3.8 L/min, cardiac index (CI) of 1.9 L/min/m^2^, stroke volume index of 32 mL/m^2^, pulmonary vascular resistance (PVR) of 3.7 WU, and pulmonary artery (PA) compliance of 2.85 mL/mm Hg (Figure 3).

## Question 1: Based on the physical examination, stress test, perfusion/Computed Tomography Angiography Scan imaging, echocardiographic findings, and hemodynamics, what would be the next step in the management of this patient?

**Figure 4:**
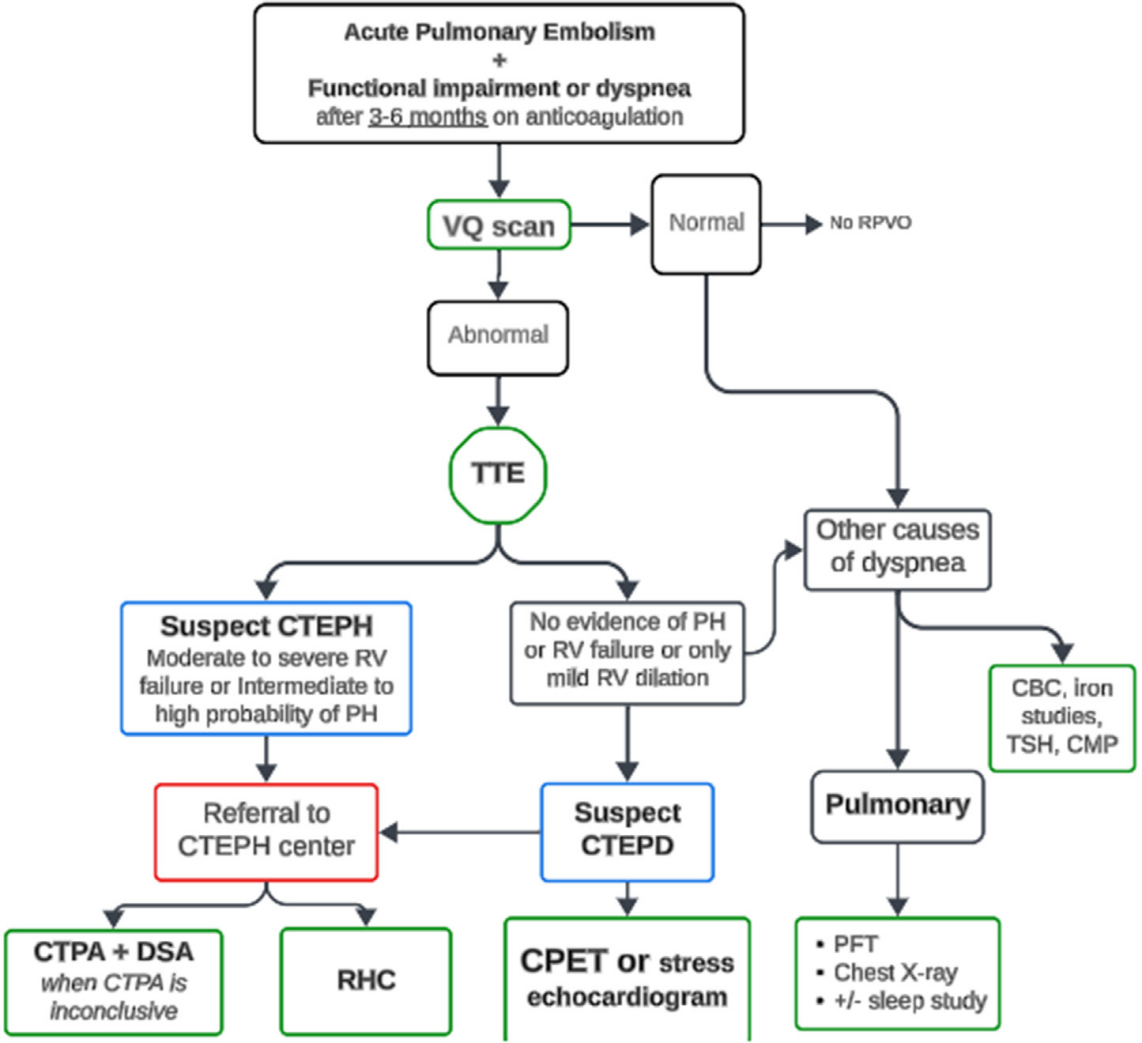
Diagnostic Algorithm for Patients with Post–Pulmonary Embolism Impairment Syndrome

This case demonstrates the presentation and evaluation of functional limitations associated with residual pulmonary vascular obstruction (RPVO) after an intermediate-risk PE in a patient without any echocardiographic or resting hemodynamic evidence of right ventricle (RV) dysfunction or significant PH (Figure 4). He underwent a right-sided heart catheterization, suggestive of mild PH by mean pulmonary artery criteria. Therefore, our concern was to evaluate the physiological impact of the RPVO on his exercise capacity. We decided to obtain an invasive cardiopulmonary exercise test (iCPET). Incorporating information from an iCPET into the algorithm before and after intervention allows us to understand the physiological etiology of dyspnea, especially when resting hemodynamics and echocardiogram are unremarkable.

On iCPET with a 10 W/min ramp protocol on a supine braked ergometer, he attained 179 W and stopped due to moderate dyspnea. The PA saturation at rest was 67% and 16% at peak exercise. His hemodynamics at peak exercise were RA of 10 mm Hg, PAP of 110/30 (57) mm Hg, pulmonary capillary wedge pressure of 15 mm Hg, CO of 11.6 L/min, CI of 6 L/min/m^2^, heart rate of 121 beats/min, PVR of 3.6 WU, and stroke volume index of 50 mL/m^2^. Gas exchange variables showed an respiratory exchange ratio of 1.18 and a peak oxygen uptake (Vo_2_) of 26 mL/kg/min. There was evidence of ventilatory inefficiency represented by an abnormal minute ventilation–carbon dioxide production ratio slope of 40 and end-tidal CO_2_ of 25 mm Hg. The oxygen saturation decreased to 85% with exertion. ΔCO/Vo_2_ was low-normal at 5. He had a normal breathing reserve of 35%, suggesting no evidence of significant pulmonary parenchymal disease.

## Question 2: What are the features in his iCPET to suggest that his dyspnea is related to CTEPD and not to other competing causes of dyspnea (eg, deconditioning, aging, subjective perception)?

The resting hemodynamics suggested mild PH by mean pulmonary artery criteria and mild elevation of PVR, with low CI. At peak exercise, there was evidence of significant exertional PH, a result of the physiological interaction of an augmenting CO with a mild but persistently elevated PVR and reduced PA compliance. It is essential to highlight the lack of PVR reduction at peak exercise, indicating an abnormal pulmonary vascular response to exercise. The gas-exchange evidence of ventilatory inefficiency indicates excess physiological dead space with exertion and parallels the lack of PVR reduction with exercise. Although stroke volume augmentation was relatively preserved with exercise, the increase in right atrial pressure from 1 to 10 mm Hg indicates a subtle disturbance in RV-PA coupling, which is only revealed with exercise. Taken together, these findings support a diagnosis of CTEPD, with aerobic exercise limitation related to hemodynamic and gas-exchange abnormalities imposed by significant RPVO. His peak Vo_2_ of 26.5 mL/kg/min is more typical of a healthy sedentary person, representing a reduction in his exercise capacity relative to his high-functioning baseline before PE.

In our case, the iCPET demonstrated an abnormal pulmonary vascular response to exercise, gas-exchange evidence of increased dead space ventilation (ventilatory inefficiency), and subtle evidence of RV-PA uncoupling only revealed during exercise testing. Although impaired exercise capacity and ventilatory inefficiency have been described in patients with chronic thromboembolic pulmonary hypertension (CTEPH) undergoing balloon pulmonary angioplasty (BPA), we highlight a specific CTEPD case without RV-PA uncoupling at rest and only subtle evidence at exertion.

## Question 3: What additional imaging could help with decision making?

**Figure 5:**
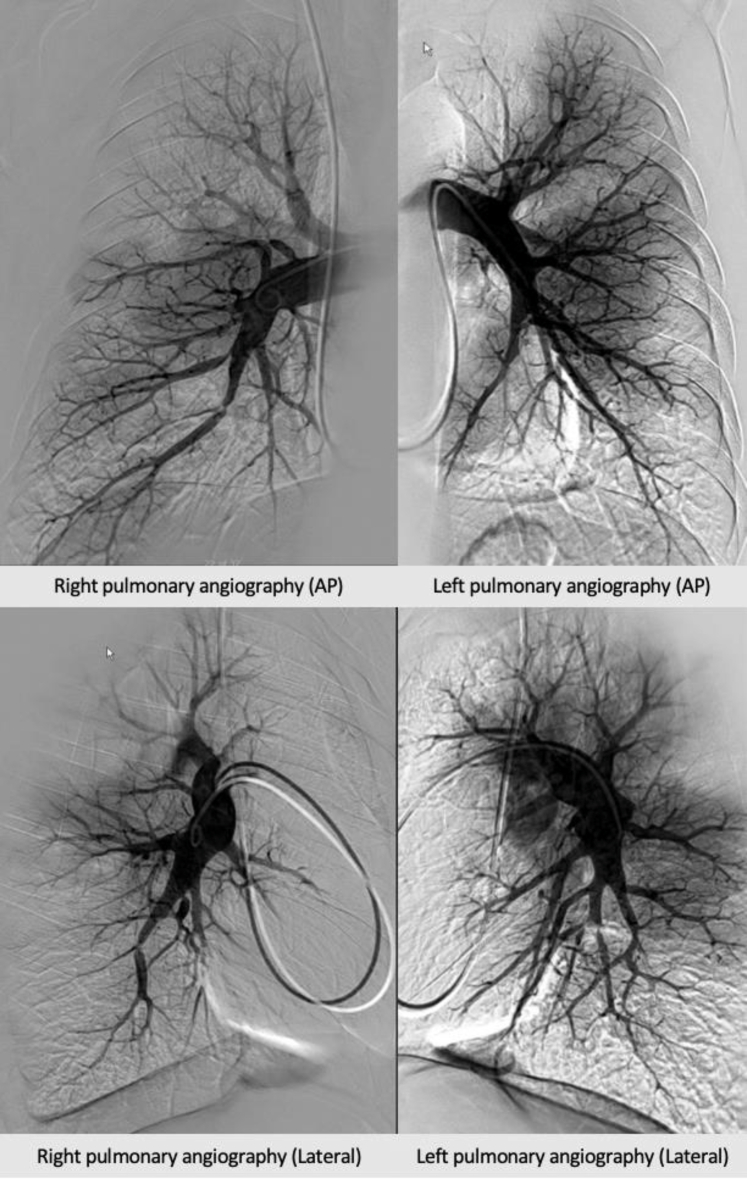
Baseline Pulmonary Angiogram

Subsequent invasive PA gram showed distal CTEPD, with high-grade stenosis (Figure 5). A multidisciplinary meeting between cardiology and cardiothoracic surgery took place.

## Question 4: What therapeutic approaches could be offered in CTEPD?

Discussions regarding the possibility of offering medical management with pulmonary vasodilators, surgical pulmonary thromboendarterectomy, or BPA took place.

The use of pulmonary vasodilators in patients with mild PH can cause more side effects than hemodynamic benefit. Pulmonary thromboendarterectomy risks (ie, bleeding, stroke, death) in a patient with distal disease would outweigh the benefits, especially in an individual without severe hemodynamic impairment. Given the segmental and subsegmental location of the chronic clot, BPA was thought to be the best option.

**Figure 6:**
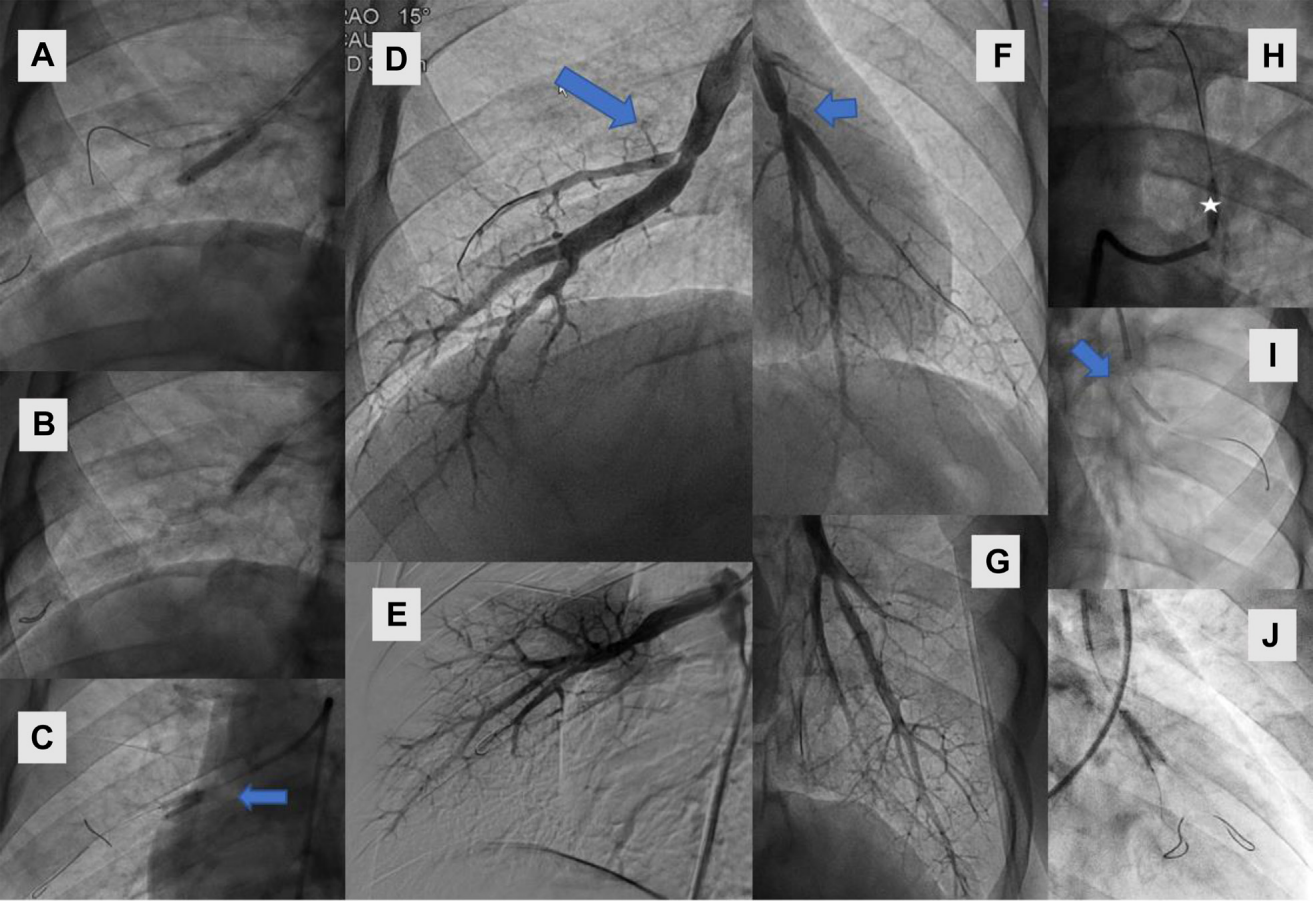
Pulmonary Angiograms and Balloon Pulmonary Angioplasty Images (A, B, C, H, I, J) Balloon inflations. (D, F) Preintervention stenotic lesions. (E, G) Postintervention. (C, J) Kissing balloon inflations at bifurcation lesions.

Given the patient’s desire to return to his prior athletic lifestyle, he underwent 4 sessions of BPA in 17 vessels, treating 33 lesions, many of which required kissing balloon inflations at the branch points (Figure 6).

## Question 5: How do you reassess outcomes after BPA in CTEPD?

**Figure 7:**
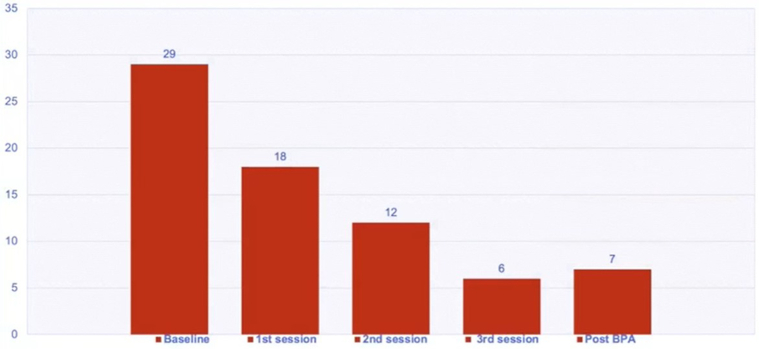
Quality of Life Scores

Three months post-BPA, repeat hemodynamic assessment revealed a PAP of 25/5 (12) mm Hg, CI of 1.8 L/min/m^2^, PVR of 2 WU, and PA compliance increase to 3.25. His 6MWD increased to 585 m (Δ54 m), V_E_/V_CO2_ slope decreased to 34, and Spo_2_ at peak exercise was 96%. His quality of life (QOL) score improved (Figure 7).

Post-BPA in CTEPD, improvements in functional capacity are primarily linked to the reduction of dead space and improvement of the PA compliance (stroke volume/PA pulse pressure), as opposed to significant changes in the relatively normal preintervention hemodynamics. The improvement in PA compliance in this case suggests a decrease of the dynamic RV afterload and thus improved cardiac efficiency and gas exchange, as demonstrated by improvements in V_E_/V_CO2_ slope, resolved hypoxia, and normal PETCO_2_. This was corroborated by restoring the alveolar blood flow after BPA, as seen on a repeat lung perfusion scan. Our case also demonstrates the potential role of BPA in treating RPVO to reduce dead space ventilation, improve oxygenation, and improve the patient’s QOL. With early intervention targeting RPVO, we can minimize the functional limitations that patients experience.

## Clinical Perspective

PE survivors frequently develop long-term exercise intolerance that markedly impairs their health-related QOL.^1^^,^^2^ RPVO accounts for more than 15% of this disability in acute PE.^3^^,^^4^ RPVO has a range of presentations. The most significant one is CTEPH, defined as PH with a resting mean PAP of >20 mm Hg secondary to chronic thromboembolic material.^2^ A more frequent cause of disability related to this chronic thromboembolic condition is called CTEPD, previously referred to as chronic thromboembolic disease. CTEPD patients do not have PH at rest but may demonstrate abnormal exercise hemodynamics and/or gas exchange parameters.

RPVO is a persistent perfusion defect on a ventilation-perfusion lung scan after 3-6 months of anticoagulation for acute PE^2^. RPVO is present in up to 30% after an acute intermediate or high-risk PE at 6 to 12 months and is a predictor of adverse clinical outcomes such as recurrent PE, CTEPD and CTEPH, heart failure, and death.^5^^,^^6^ This review presents a case-based approach to evaluate the functional limitations due to RPVO after acute PE.

In conclusion, RPVO may be a significant cause of post-PE syndrome in intermediate- and high-risk PE patients. An echocardiogram and resting invasive hemodynamics may be insufficient in elucidating abnormalities in CTEPD, and 6MWDT may not provide a clear assessment of the functional status. Current guidelines recommend CPET when there is an intermediate echocardiographic probability of PH.^7^ However, CPET, particularly iCPET, can give a more comprehensive evaluation of the physiological causes of dyspnea in this population and should be considered even without echocardiographic or resting hemodynamic evidence of RV dysfunction or significant PH. Therefore, early referral to CTEPH centers of excellence is recommended.

## Funding Support and Author Disclosures

This research was funded by the Division of Cardiovascular Disease, Temple University Hospital. Dr Bashir has been funded by NHLBI; and has an equity interest in Thrombolex Inc. Dr Oliveros has received a research grant from Johnson and Johnson. All other authors have reported that they have no relationships relevant to the contents of this paper to disclose.
